# A Globe Pessary Worn 35 Years without Removal

**Published:** 1882-02

**Authors:** A. Reeves Jackson

**Affiliations:** Chicago, Ill.


### CLINICAL REPORTS.

### A GLOBE PESSARY WORN 35 YEARS WITHOUT REMOVAL.

By A. REEVES JACKSON, A. M., M D., Chicago, Ill.

In the winter of 1869, while living at Stroudsburg, Pa., I visited E. B., an unmarried woman 50 years of age, a resident of the same place, and for whom I had during a number of years prescribed occasionally for various ailments, chiefly of a nervous and dyspeptic character. Never before, however, had I been acquainted with her full history.

Endowed from the first with a feeble organization, she was of spare habit and sallow complexion. Menstruation commenced at 13, was always painful, and for the first few years scanty. At the age of 15 she visited Philadelphia and consulted the late Prof. Chas. D. Meigs, who placed in the vagina a globe pessary, and instructed the patient to remove it at intervals of two or three weeks—furnishing her at the same time with a scoop-like instrument, made of bent wire for this purpose. Accordingly, she subsequently made a few painful and unsuccessful attempts to carry out this direction. However, as she had no suffering from the presence of the instrument she finally abandoned all further efforts to remove it.

A leucorrhœal discharge—at first yellowish-white in color and unirritating in character, and becoming afterwards yellowish-brown and acrid, was constantly present. She likewise suffered from occasional pelvic and lumber pains, headache, gastralgia, nervousness and irritability.

From the combined influence of mal-nutrition, sleeplessness and pain, she at last became a chronic invalid. At about the age of 43, menstruation appeared at shorter intervals and rather profusely. This was attributed to the "change of life," and no medical advice was obtained. By-and-by, metrorrhagia set in, and the patient was rarely free from a more or less copious bloody discharge. The bowels were constipated and the bladder was at times irritable.

On examination, I found the entrance to the vagina obstructed by a hard substance, of which only a portion, about the size of a dime could be felt, the remainder of the foreign body being so closely grasped by the vagina that a finger could not be inserted between the two. An attempt to do so was accompanied by so much pain and hemorrhage that I determined to administer chloroform before proceeding further. This was accordingly done, and after considerable difficulty I succeeded in separating the pessary from the vagina by passing a finger entirely around the former. I then introduced the wire scoop far enough to embrace the upper anterior segment of the sphere and, by strong traction, aided by the downward and forward pressure of a finger the pessary was finally delivered. The operation occupied about twenty minutes, and was attended by the loss of a good deal of blood.

The pessary, which I exhibited at a meeting of the Chicago Medical Society some years ago, consists of a hollow globe apparently of gold, 1⅝ inches in diameter. When first removed its entire surface, except the small part which was not embraced by the vagina, was incrusted with a black deposit of considerable thickness, which I removed by filing and sand-papering, thus exposing the bright golden surface beneath.

The patient soon recovered from the operation and in a few days the hemorrhage and leucorrhœa disappeared, these discharges having evidently been continued by the presence of the foreign body.

